# Endoscopic Management of Pancreatic Fluid Collections

**DOI:** 10.3390/jcm10020284

**Published:** 2021-01-14

**Authors:** Robert Dorrell, Swati Pawa, Rishi Pawa

**Affiliations:** 1 Department of Medicine, Wake Forest School of Medicine, Winston-Salem, NC 27157, USA; rdorrell@wakehealth.edu; 2 Department of Medicine, Section of Gastroenterology, Wake Forest School of Medicine, Winston-Salem, NC 27157, USA; spawa@wakehealth.edu

**Keywords:** pancreatic fluid collection, lumen-apposing metal stent, direct endoscopic necrosectomy, necrotizing pancreatitis, pseudocyst, dual-modality drainage, disconnected duct syndrome, multiple transluminal gateway technique

## Abstract

Pancreatic fluid collections (PFCs) are a common sequela of pancreatitis. Most PFCs can be managed conservatively, but symptomatic PFCs require either surgical, percutaneous, or endoscopic intervention. Recent advances in the therapeutics of PFCs, including the step-up approach, endoscopic ultrasound-guided transmural drainage with lumen apposing metal stents, and direct endoscopic necrosectomy, have ushered endoscopy to the forefront of PFCs management and have allowed for improved patient outcomes and decreased morbidity. In this review, we explore the progress and future of endoscopic management of PFCs.

## 1. Introduction

Pancreatic fluid collections (PFCs) are a consequence of pancreatic injury from acute or chronic pancreatitis, surgery, or trauma. These collections form due to the release of proteolytic fluid from the pancreas into the adjacent cavity of the peritoneum. Most PFCs are asymptomatic and rarely require invasive intervention; however, when PFCs cause symptoms, including abdominal pain, nausea, and vomiting secondary to gastric outlet obstruction, biliary obstruction, or fever secondary to an infection, drainage is indicated [1]. Techniques to drain PFCs have evolved from percutaneous and invasive surgical necrosectomy to direct endoscopic necrosectomy as endoscopic technology has advanced. The novel endoscopic approach has drastically improved patient morbidity and mortality [2]. This review explores the role of endoscopy in the management of PFCs.

## 2. Classification of Pancreatic Fluid Collections

Both the diagnosis and management of PFCs have improved over the last several decades. Prior to advanced imaging techniques like cross-sectional imaging and ultrasound, PFCs were primarily identified through palpable abdominal masses on physical exam and displacement of the stomach on barium swallow. Any patients with suspected symptomatic PFCs required an exploratory laparotomy for surgical drainage to minimize the risk of hemorrhage and rupture. This approach led to unnecessary procedures and significant morbidity and mortality. Fortunately, the management of PFCs patients improved with the advent of advanced imaging techniques that allow for enhanced visualization of the pancreas. For example, in the 1970s, transabdominal ultrasound emerged as a new modality to identify PFCs. This innovation allowed physicians to identify PFCs more accurately and take a more conservative approach with regards to surgical interventions [3,4].

In 1992, the Atlanta classification system provided a method to categorize PFCs. This classification system has been instrumental in guiding both diagnosis and treatment. The Atlanta classification system has remained relevant to the management of PFCs because it has been modified as PFC pathophysiology has become better characterized [5]. In 2012, this classification system was revised to include four types of PFCs: acute peripancreatic collections, pancreatic pseudocysts, acute necrotic collections (ANCs), and walled-off pancreatic necrosis (WOPN). Additionally, this updated classification system recommended intervention based on symptoms rather than size [6]. The 2012 revised Atlanta classification system remains the cornerstone for the management of PFCs today (Table 1).

The type of PFC, as defined by the Atlanta classification system, helps direct its management. The four collections differ in acuity and the presence of necrosis. PFCs without necrosis include acute peri-pancreatic fluid collections and pancreatic pseudocysts. Acute peri-pancreatic fluid collections present less than four weeks after the onset of acute pancreatitis (AP) [6]. Due to their acute nature, these PFCs lack a mature capsule and are typically not amenable to endoscopic intervention. However, they rarely require intervention as the majority resolve spontaneously [7]. On the other hand, pancreatic pseudocysts, which occur more than four weeks after AP, form a mature capsule and are amenable to endoscopic drainage. PFCs with necrotic material include ANCs, which occur within four weeks of AP, and WOPN, which occur more than four weeks after AP. In the same fashion as the non-necrotic PFCs, ANCs are usually not amenable to endoscopic drainage due to the lack of a mature capsule. In contrast, WOPN can be drained due to wall maturity. 

In addition to acuity and the presence of necrosis, the risk of infection is another defining characteristic of PFCs. Infected PFCs are particularly important to identify because they are associated with an increased mortality rate of up to 30% in comparison to non-infected WOPN, which carry a mortality rate of 10% [8]. Various imaging modalities can be used for this purpose [6,9]. Computed tomography (CT) of the abdomen has a sensitivity of 56% and specificity of 97% for identifying an infected PFC by the presence of air within the collection [10,11]. Another modality for identifying an infected collection is the fine-needle aspiration of the PFC. This method has an increased sensitivity of 79% but is not routinely used due to its low diagnostic yield and high false-negative rate [12]. There are currently two schools of thought regarding immediate versus postponed percutaneous drainage for the management of infected WOPN. The POINTER trial is a randomized controlled trial that attempts to compare these two approaches in order to identify the best method to manage acutely infected PFCs [13]. The results of this trial were presented at the United European Gastroenterology Week in October 2020, and no differences existed between the two groups regarding mortality, new, onset organ failure, or other major complications. However, the median number of interventions was significantly higher in the immediate catheter drainage group (*p* < 0.001).

## 3. Current Approach to Management of Infected Pancreatic Fluid Collections

While the POINTER trial is set to greatly impact PFC management, the current approach to the management of infected PFCs is based on three landmark publications. The PANTER trial published by the Dutch Pancreatitis Study Group first suggested a step-up approach to infected PFCs in 2010. Santvoort et al. published a randomized control trial (PANTER) in which patients with infected PFCs were either randomized for open necrosectomy or a step-up approach. Patients in the step-up arm were managed with minimally invasive techniques like percutaneous or endoscopic transgastric drainage with escalation to minimally invasive retroperitoneal necrosectomy if there was no clinical improvement. The primary outcomes included major complications (organ failure, perforation, fistula, and bleeding) and mortality. The step-up approach demonstrated significantly fewer major complications (risk ratio 0.57; 95% [0.38–0.87] *p* = 0.006), decreased healthcare utilization (*p* = 0.004), and no significant difference in mortality (19% vs. 16% *p* = 0.002) [14]. This study not only solidified the step-up approach as the standard of care for infected PFCs, but it showcased the utility of minimally invasive interventions for this disease process. 

The PANTER trial was soon followed by the PENGUIN trial in 2012. This randomized trial directly compared endoscopic necrosectomy and surgical necrosectomy in 20 patients by assessing postprocedural inflammation with interleukin-6 (IL-6) levels and major complications. Endoscopic necrosectomy had lower levels of postprocedural IL-6 (*p* = 0.004) and fewer major complications (20% vs. 80%; risk difference 0.60, 95% CI 0.16–0.80, *p* = 0.03) compared to the surgical approach. These findings were confirmed in numerous retrospective cohorts including a multicenter study by the German pancreatitis study group in 2016, which showed a reduction in mortality for endoscopic necrosectomy in comparison to open surgical necrosectomy (10.5% vs. 33.3%) [15].

In 2017, the Dutch pancreatitis study group performed a randomized controlled trial (TENSION) to compare the surgical step-up approach to the endoscopic step-up approach for the management of infected necrotizing pancreatitis. The surgical step-up approach included percutaneous drain placement in the left retroperitoneal cavity followed by escalation to video-assisted retroperitoneal debridement through the existing tract if needed. The endoscopic step-up group consisted of the placement of two 7-French double-pigtail stents along with a nasocystic catheter with endoscopic transluminal necrosectomy performed in patients who lack clinical improvement. This study found no significant difference in mortality but showed a decreased rate of pancreatic fistula formation and reduced hospital length of stay in the endoscopy group [16].

## 4. Endoscopic Ultrasound-Guided Transmural Drainage Versus Conventional Transmural Drainage

**Figure 1:**
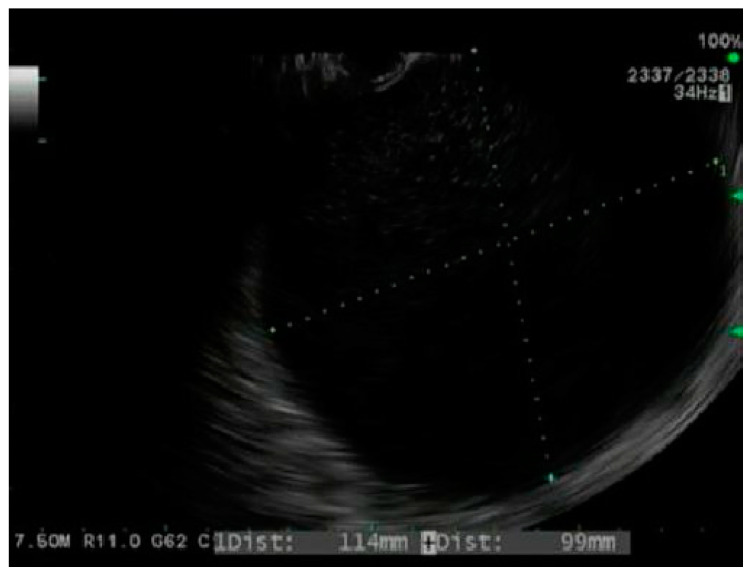
Endoscopic ultrasound image of a pseudocyst.

Endoscopic drainage of PFCs was first reported in 1989 with the conventional transmural drainage (CTD). This process involved endoscopic visualization of a bulge in the gastric or duodenal wall secondary to compression by a PFC [17]. The collection was punctured through the gastric wall to create a cystgastrostomy tract, followed by tract dilation and plastic stent placement via the Seldinger technique. While this technique is safe and effective, it is limited because only 40–50% of PFC patients have a physical gastric or duodenal bulge to guide tract creation [18]. Endoscopic ultrasound-guided transmural drainage (EUS-TD) allows for direct visualization of the PFC, identification of intervening blood vessels or necrosis, and aspiration of PFC fluid to confirm placement within the collection (Figure 1) [19]. Kahaleh et al. compared the effectiveness and complication rate of CTD to EUS-TD and found no significant difference [20]. However, CTD was only performed for patients with a visible gastric protrusion, whereas EUS-TD was performed on those without. Therefore, EUS-TD was mostly used on patients with smaller collections, which are typically more difficult to drain than larger collections. Varadarajulu et al. also compared EUS-TD and CTD in a prospective randomized trial of 30 patients with PFCs. They reported a clinical success rate of 100% for EUS-TD versus 33% for CTD [21]. Furthermore, in a prospective randomized trial, Park et al. found that EUS-TD had fewer complications and a higher success rate in non-bulging cysts. Therefore, EUS-TD should be employed over CTD for endoscopic drainage of PFCs [22]. 

## 5. Types of Stents for Drainage of Pancreatic Fluid Collections

Just as EUS-TD has improved the safety and efficacy of PFC drainage, so have metal stents. Historically, plastic stents (PS) were the mainstay of PFC drainage. However, their placement can be time-consuming and challenging because multiple stents are required to properly drain PFCs with PS. As a result, fully covered self-expandable metal stents (FCSEMSs) became increasingly popular due to their large diameter and ease of placement. In a prospective case series of 18 patients, Talreja et al. demonstrated the safety and efficacy of FCSEMSs for drainage of PFCs. Ninety-five percent of the collections were successfully drained; stent migration was seen in one patient, post stent placement bleeding in two patients, and superinfection in five patients [23]. These findings were also demonstrated by Penn et al. [24] and Sarkaria et al. [25], who found FCSEMSs to be effective for drainage of PFCs.

**Figure 2:**
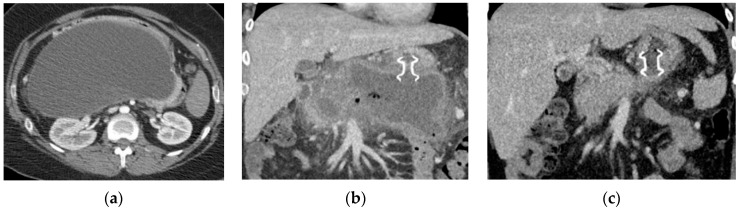
Computed tomography (CT) imaging of pancreatic pseudocyst. (**a**) Sagittal CT pancreas protocol image of a large 14.7 cm × 19.3 cm pseudocyst prior to lumen-apposing metal stent (LAMS) placement; (**b**) coronal CT image of pancreatic pseudocyst one day post-LAMS placement; and (**c**) coronal CT image of pancreatic pseudocyst six weeks post-LAMS placement.

While luminal metal stents greatly improved PFC drainage, the risk of migration and injury to the opposing luminal walls (gastric wall, duodenal wall, and retroperitoneum) prevented their widespread use. The development of the lumen-apposing metal stents (LAMSs) has revolutionized the endoscopic management of PFCs. LAMSs are advanced biflanged-shaped metal stents designed to minimize the risk of stent migration (Figure 2). Furthermore, the large diameter of this dumbbell-shaped stent allows for the passage of an endoscope inside the cyst cavity to perform direct endoscopic necrosectomy (DEN). Additionally, LAMS variants like the HOT AXIOS stent (Boston Scientific, Marlborough, MA, USA) have a built-in electrocautery tip, which allows for rapid deployment of the stent without the use of a wire and greatly reduces procedure time. Numerous studies have evaluated the efficacy of LAMS for PFCs compared to PS. In a study by Tan et al., comparing PS to LAMS in 875 PFC patients (pseudocysts and WOPN), LAMSs were associated with lower complication rates and higher clinical success rates. The increased success of LAMS was attributed to its larger diameter; however, this feature also increased the risk of bleeding (*p* ≤ 0.001) due to stent friction against blood vessels and increased volumes of gastric fluid entering the cyst cavity [26]. These findings were corroborated in a study by Hammad et al., which also concluded that LAMSs were associated with better clinical outcomes; however, there were fewer overall adverse events than PS in this study [27]. In an international multicenter comprehensive analysis of adverse events with LAMS for drainage of PFCs, Fugazza et al. confirmed the previously demonstrated high technical and clinical success but recommended future prospective studies to further evaluate their safety [28].

## 6. Endoscopic Management of Pseudocysts

**Figure 3:**
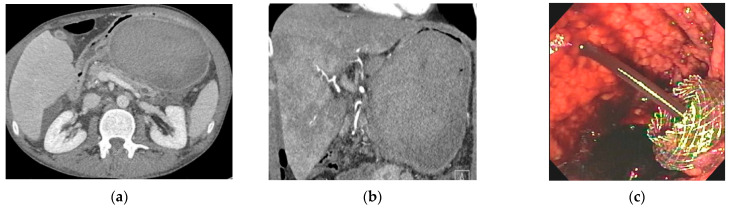
CT and endoscopic image of a hemorrhagic pseudocyst. (**a**) Sagittal CT imaging of pseudocyst with heterogenous material consistent with hemorrhage; (**b**) coronal CT imaging of hemorrhagic pseudocyst prior to LAMS placement; and (**c**) endoscopic imaging post-LAMS placement with blood tinged fluid draining in the stomach.

While general guidelines support the use of larger stents like LAMSs and the step-up approach for drainage of PFCs, the treatment algorithm becomes more specialized based on the type of PFC. Pseudocysts are mature fluid collections without the presence of necrosis (Figure 3). The symptoms from pseudocysts are largely caused by a local mass effect from the cyst on adjacent organs or infection. Once a patient is symptomatic, they should be evaluated for drainage. 

The endoscopic management of pseudocysts is controversial because few studies have focused solely on this entity. Aburajab et al. compared the safety and efficacy of LAMS to LAMS plus double-pigtail stents (DPSs) for pseudocysts and found that LAMSs alone were associated with higher rates of post-procedural infection, reintervention, and nonresolution (Figure 3). In the LAMS-alone group, 91% of patients had cyst resolution and 17% had post-operative infections. In the LAMS with DPS group, 100% of patients saw cyst resolution and no patients experienced an infection. The authors thus concluded that DPS should be placed across the LAMS at the index procedure for pseudocyst drainage to improve clinical success and reduce the risk of infection [29].

Yang et al. in a large multicenter study compared the role of LAMS to PS in the management of pseudocysts. They found no significant difference in technical success, recurrence rate, or post-procedural length of stay. However, LAMS was associated with higher clinical success (96.3% vs. 87.2%; *p* = 0.03) and decreased need for follow-up percutaneous procedures (1.3% vs. 4.9%; *p* = 0.04) [30]. While LAMSs have shown success in the management of pseudocysts, their novelty also comes with increased cost. Chen et al. performed an analysis of LAMS vs. PS and found LAMS to be significantly more expensive without any added benefit, and thus concluded that PSs are preferred over LAMSs for management of pseudocysts [31].

## 7. Endoscopic Management of Walled-Off Pancreatic Necrosis

Endoscopic management of WOPN is more challenging than pseudocysts given the presence of solid necrotic material that makes drainage with standard endoscopic techniques more difficult. Siddiqui et al. performed a retrospective analysis of 313 patients to compare the three different types of stents for the management of WOPN—LAMSs, PSs, and FCSEMSs. They found that for WOPN, FCSEMSs and LAMSs were superior to PSs in clinical outcomes and that LAMSs had lower reintervention rates than both PSs and FCSEMSs [32]. While this study showed the superiority of LAMSs over PSs in the management of WOPN, these results were not replicated in a randomized controlled trial of 60 patients by Bang et al. [33]. The authors found no statistically significant difference for any outcome between the two groups (PSs vs. LAMSs); however, given the small sample size, the study likely did not have enough power to achieve significant findings. 

While no large, randomized studies have been performed comparing PSs and LAMSs for management of WOPN, a retrospective large international multicenter study by Chen et al. demonstrated the superiority of LAMSs over PSs for endoscopic drainage of WOPN in 189 patients. LAMSs were associated with higher clinical success rates, shorter procedure time, lower need for surgical intervention, and lower reintervention rates [34]. To ensure safe LAMS deployment, a diagnostic evaluation with EUS is required in order to measure the precise thickness of the PFC capsule wall. This can sometimes be challenging in patients with infected hemorrhagic collections using the B-mode EUS imaging. Contrast-enhanced harmonic endoscopic ultrasonography (CH-EUS) is a useful modality that can allow for precise measurement of the PFC capsule wall and potentially improve the safety of LAMS deployment [35]. Furthermore, developments in the LAMS stents has increased their stent diameter from 10 mm and 15 mm to 20 mm. Parsa et al. compared the effectiveness of 20 mm vs. 15 mm LAMSs for WOPN. They demonstrated that 20 mm LAMSs had similar clinical success and adverse event rates as compared to 15 mm LAMSs; however, fewer endoscopic necrosectomies were needed for WOPN resolution [36]. In conclusion, both diameter stents are safe and effective for WOPN; however, collections with extensive necrosis may benefit from the placement of larger diameter stents. 

## 8. Direct Endoscopic Necrosectomy

The presence of necrosis in PFCs increases patient mortality and often requires direct endoscopic necrosectomy to facilitate optimal drainage. WOPN is particularly susceptible to infection, which can cause systemic complications including sepsis and organ failure. Infected WOPN is associated with longer hospitalizations, an increased number of percutaneous procedures, and 40% mortality [37]. Traditionally, these collections were managed with percutaneous drains with escalation to open surgical necrosectomy if necessary. Over the last decade, DEN has evolved into the preferable approach because it is associated with decreased patient morbidity. 

**Figure 4:**
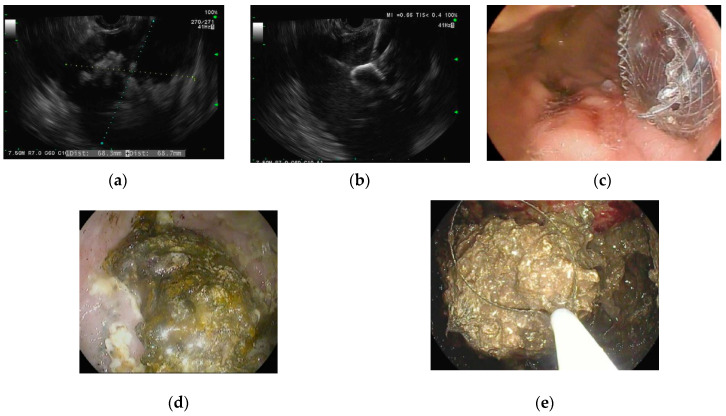
Endoscopic ultrasound-guided drainage using a LAMS with subsequent direct endoscopic necrosectomy for walled-off pancreatic necrosis (WOPN) (**a**) Endoscopic ultrasound imaging of cyst cavity with necrotic debris; (**b**) endoscopic ultrasound-guided placement of LAMS into WOPN; (**c**) endoscopic imaging post LAMS deployment in the stomach; (**d**) endoscopic imaging inside cyst cavity with necrotic material; and **(e)** direct endoscopic necrosectomy performed using a snare.

DEN involves the passage of an endoscope from the gastrointestinal tract into the necrotic cavity adjacent to the gastric or duodenal wall followed by debridement of necrotic material from the cavity with snares, forceps, and baskets (Figure 4). This is the preferred treatment modality for WOPN due to its low complication rates, decreased length of hospital stay, and high clinical success rates [38]. The procedure was first described in a case series of 11 patients by Baron et al. The use of DEN avoided further surgical intervention in nine out of the 11 patients, which was viewed as a tremendous success for WOPN management [39]. Seifert et al. later performed a multicenter trial (GEPARD) in which 93 patients with WOPN treated with DEN were followed up for understanding the treatment’s long-term outcomes. This trial reported an 80% clinical success rate, 26% complication rate, and a 7% mortality rate after 30 days. The patients were followed up with for about four years, and over this time period, 84% achieved resolution of the collection, with 10% requiring additional endoscopic procedures and 4% requiring surgical intervention [40]. The GEPARD study positioned DEN as an essential procedure for the management of WOPN [15]. One limitation of DEN is the lack of appropriate tools to perform a necrosectomy efficiently. Over the last few years, novel endoscopic morcellator devices have been developed to facilitate this procedure [41]. These tools have a rotating blade that can cut, suction, irrigate, and collect specimens, thereby successfully liquifying and removing solid necrosis from the walled-off cavity [42]. In a case series of 12 patients by Van der Wiel et al., a total of 27 procedures were performed with no adverse events [43]. Prospective trials are underway to evaluate the safety and efficacy of this new device. Improved methods of debridement with such devices can further reduce the need for surgical necrosectomy for WOPN.

## 9. Dual-Modality Drainage

Dual-modality drainage is a combined percutaneous and endoscopic approach that intends to reduce the risk of pancreaticocutaneous fistulae and avoid open surgical necrosectomy. This approach was first introduced at Virginia Mason Medical Center in 2014 in response to the high rate (20%) of chronic pancreaticocutaneous fistulae in patients with disconnected duct syndrome [44,45]. During this procedure, placement of percutaneous drains is immediately followed by endoscopic placement of transluminal stents in an effort to drain pancreatic juice into the gastrointestinal tract rather than through the skin. In a prospective study of 117 patients, Ross et al. found that the rate of pancreaticocutaneous fistulae dropped from 20% for PFCs with percutaneous drainage alone to 0% for dual-modality drainage [44]. This study was limited by the lack of a comparison group undergoing endoscopic drainage alone; however, the authors still concluded that dual-modality drainage had a lower complication rate compared to the rates reported in separate studies of endoscopic drainage with DEN. In a study by Yokoi et al., patients with pancreatic duct disruption were noted to have PFCs with high amylase content (>10,000 U/L) and responded well to dual-modality drainage (percutaneous drainage combined with transpapillary nasopancreatic drainage) [46]. Further prospective studies are needed to evaluate the efficacy of dual-modality drainage in comparison to endoscopic therapy alone. 

## 10. Disconnected Duct Syndrome

**Figure 5:**
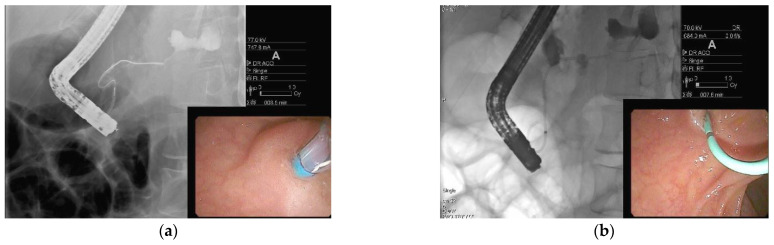
Endoscopic retrograde pancreatography in a patient with disconnected duct syndrome (DDS). (**a**) Pancreatogram showing extravasation of contrast from the pancreatic duct in the body of the pancreas. The pancreatic duct in the tail is not opacified. The above findings are suggestive of complete DDS; and (**b**) pancreatic sphincterotomy followed by pancreatic duct stent placement.

Thus far, this review has focused on modalities to drain PFCs and remove necrotic debris, but a third essential goal of endoscopic management is to prevent PFC recurrence. Disconnected duct syndrome (DDS) is the most common cause of PFC recurrence due to necrosis and structural disintegration of the pancreas (Figure 5) [47]. The disruption of the main pancreatic duct (MPD) prevents secreted pancreatic juices from draining into the duodenum. The duct disruption can be partial (marked by the ability to opacify the MPD upstream past the disruption on endoscopic retrograde pancreatography (ERP)) or complete (marked by the inability to opacify the MPD upstream past the disruption on ERP) [48,49].

**Figure 6:**
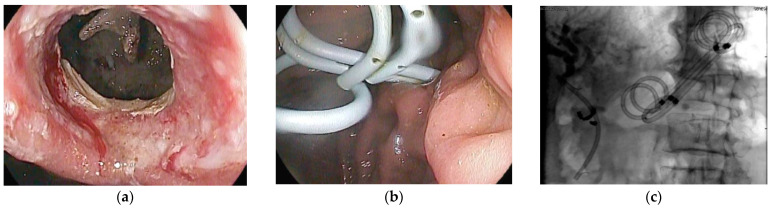
Disconnected duct syndrome with LAMS removal and placement of double pigtail stents. (**a**) Endoscopic imaging showing LAMS removal with a residual portal into cyst cavity in a patient with known disconnected duct syndrome; (**b**) transmural plastic double-pigtail stents placed inside the cyst cavity; (**c**) fluoroscopic image showing double-pigtail stents to prevent recurrence of pancreatic fluid collection.

DDS first came to light in the 1990s, but its prevalence was initially underestimated given that it was best diagnosed with ERP and had no radiologic diagnostic criteria [50]. Maatman et al. performed the largest retrospective review of patients with necrotizing pancreatitis (*n* = 647) and found a DDS prevalence of 36%. DDS was associated with worse outcomes and increased rates of PFC recurrence [51]. While surgery has long been the cornerstone of DDS management, advances in endoscopy have provided minimally invasive alternatives that will likely minimize the role of surgical intervention in the future. Endoscopic management of DDS includes transpapillary drainage (Figure 6), which involves placing a plastic stent into the main pancreatic duct to allow the pancreatic juices to flow into the duodenum [52]. Jang et al. found transpapillary drainage alone was 92% effective in partial pancreatic duct disruptions, but only 20% effective in complete pancreatic duct disruptions. In addition, patients with complete pancreatic duct disruptions were more likely to undergo surgical management [49].

Tellez-avina et al. studied the effectiveness and safety of permanent indwelling transmural stents for DDS in 21 patients and found them to be safe and efficacious [53]. Bang et al. also evaluated endoscopic management of DDS with a study of DDS patients who underwent initial drainage with LAMS. The LAMS were replaced with double-pigtail plastic stents once the PFC resolved. Only 1.4% of patients had recurrence (*p* = 0.001) [54]. This study demonstrates that endoscopic management of DDS patients should include permanent transmural double pigtail stents placement (Figure 6) [55,56,57].

## 11. Multiple Transluminal Gateway Technique

Multiple transluminal gateway technique (MTGT) is a drainage strategy that uses numerous transmural tracts formed under EUS guidance between the GI tract and the necrotic collection. One tract is used to flush the cavity while the other tract is used to drain the necrotic contents. Varadarajulu et al. introduced MTGT using plastic stents and compared this method to conventional drainage. In a cohort of 60 patients, 12 underwent MTGT and 48 underwent conventional endoscopic drainage. They showed that MTGT was more likely to result in treatment success (adjusted odds ratio 9.24, 95% confidence interval 1.08–79.02, *p* = 0.04) and less likely to result in surgical necrosectomy [58]. They presented MTGT as an effective and viable management tool for management of WOPN. Furthermore, Bang et al. presented a successful case of MTGT using Axios stents. Using the single-step creation of tracts with Axios stents, MTGT has the potential to become essential to the framework of endoscopic management of WOPN [59,60,61]. However, larger, randomized studies are needed to validate these findings as limited studies are currently available.

## 12. Conclusions

Management of pancreatic fluid collections (PFCs) has undergone tremendous innovation over the last several decades due to cutting-edge endoscopic techniques. Endoscopy is the preferred minimally invasive treatment modality currently available for drainage of PFCs. The novel lumen-apposing metal stent (LAMS) has been shown to provide safe and effective drainage of PFCs and allows for direct endoscopic necrosectomy, which reduces the need for surgical necrosectomy. Prospective randomized controlled trials are needed to further delineate the optimal timing of endoscopic necrosectomy in the course of PFCs, duration of stent placement, and treatment of disconnected duct syndrome (DDS). This field will likely continue to grow in the future as innovative devices are currently in development to further assist with necrotic debridement.

## Figures and Tables

**Table 1:** The 2012 Atlanta classification system of pancreatic fluid collections (PFCs) [6].

Type of Collection	Age of Collection	Presence of Necrosis
Acute peripancreatic fluid collection	≤4 weeks	No
Pseudocyst	>4 weeks	No
Acute Necrotic Collection	≤4 weeks	Yes
Walled-off Necrosis	>4 weeks	Yes
